# Editorial: Respecting Human Autonomy through Human-Centered AI

**DOI:** 10.3389/frai.2021.807566

**Published:** 2021-11-26

**Authors:** Kaisa Väänänen, Supraja Sankaran, Marisela Gutierrez Lopez, Chao Zhang

**Affiliations:** ^1^ Faculty of Information Technology and Communication Sciences, Tampere University, Tampere, Finland; ^2^ Industrial Design, Eindhoven University of Technology, Eindhoven, Netherlands; ^3^ School of Sociology, Politics and International Studies, University of Bristol, Bristol, United Kingdom; ^4^ Department of Psychology, Utrecht University, Utrecht, Netherlands

**Keywords:** aritifical intelligence, human—agent interaction, autonomy, human-centerd approach, multidisciplinary approach

The past decade has seen exponential advancement in artificial intelligence (AI) in various domains. Technologies are gaining *intelligence*, becoming increasingly autonomous and are endowed with increased decision-making capabilities. These range from advanced technologies such as autonomous cars, drones, humanoid robots to a variety of systems we interact with every day, such as voice agents and social media or entertainment apps. There are numerous advantages of the advanced capabilities of autonomous intelligent systems such as automating redundant tasks, supporting better personalization, and enhancing predictions, and offering decision support.

Nonetheless, in certain contexts, these technologies also pose a threat to human autonomy by over-optimizing the workflow, hyper-personalization, or by not giving users sufficient choice, control, or decision-making opportunities. Additionally, they raise ethical challenges such as a lack of transparency and accountability owing to their fundamental black-box nature. These lead to a conundrum on how to tackle the friction between human and machine autonomy as autonomous intelligent technologies get more embedded and pervasive in our everyday lives.

Researchers working on human-centered AI have been developing models and methods to achieve fair, transparent, and accountable AI technologies using explainability, glass-box ML models, and other user-centric approaches. However, there is still a gap in identifying approaches that could enable us to develop AI-based technologies without jeopardizing human control, agency, and autonomy. It also remains unclear as to how the tension between human and machine autonomy varies across different application contexts and how the tension is viewed by researchers from different domains (e.g., computer science, philosophy and ethics, psychology, social sciences, human-computer interaction, etc.).

This collection of articles is an extended contribution of the international workshop held at the NordiCHI Conference in 2020 on the same research topic (workshop website). It expands the notion of autonomy by bringing forward perspectives from various domains such as—human-robot interaction (A6), clinical decision-support systems (A4), home automation (A7), autonomous management of drones (A3) to everyday applications of AI (A5). Additionally, it offers insights into challenges to human autonomy in AI from a regulatory perspective (A2) and through a philosophical account (A1).

Below we introduce the readers to each contribution briefly, starting from domain-general conceptual analyses to domain-specific empirical studies.

(A1) *AI Systems and Respect for Human Autonomy* *.* In this contribution, philosophers Laitinen and Sahlgren proposed a multi-dimensional model of human autonomy and discussed how AI systems might enhance or diminish autonomy based on the model. They concluded that although AI systems are not moral agents, they are expected to be designed according to autonomy-related norms and by designers who bear responsibilities. Their paper also provides a good overview of the philosophical literature on human autonomy.
(A2) *Governing AI in Electricity Systems: Reflections on the EU Artificial Intelligence Bill* . AI not only influences individuals’ everyday decisions but also critical community-level decisions such as the management of power systems. Niet et al. analyzed the recent Artificial Intelligence Act by the European Commission and identified human autonomy as one of the risks that were not adequately addressed by the Act. Their paper is a wake-up call for researching AI and autonomy issues in electricity systems.
(A3) *Human Autonomy in Future Drone Traffic: Joint Human–AI Control in Temporal Cognitive Work* . In the application domain of drone traffic control, Lundberg et al. looked at the tension between human operators and AI automation. Based on cognitive control theory, they proposed a Joint Control Framework that allows analyses of human-AI communication at different levels of autonomy and in temporal development. Their work highlights the trade-off between work efficiency and meaningfulness.
(A4) *Respecting Human Autonomy in Critical Care Clinical Decision Support* *.* Clinical decision support (CDS) systems can be autonomy-restricting for human physicians. In their conceptual analysis paper, Hendriks et al., argued for a different viewpoint that regardless of whether human physicians’ decisions are altered by CDS, their autonomy is retained if the decisions are in line with the goals and values of them and their patients. This argument led to a promising research agenda on value aware CDS.
(A5) *Exploring Peoples’ Perception of Autonomy and Reactance in Everyday AI Interactions* . In their empirical work, Sankaran et al., studied in an online experiment whether two specific factors influenced perceived autonomy in everyday human-AI interactions: receiving explanations from the system and being aware of AI used in the system. Their results suggest that the effects of these two factors are application dependent. For example, automated decisions in social media are perceived as particularly autonomy-threatening and providing the “why” of decisions protects autonomy in the context of car navigation.
(A6) *Trust Dynamics and Verbal Assurances in Human Robot Physical Collaboration* . Trust is an important autonomy-related issue to consider in human-AI interactions. Alhaji et al. examined the factors that influence human trust in physical human-robot collaboration in a lab experiment. Their results revealed a crucial distinction between trust accumulation and trust dissipation: humans are influenced by different factors when forming trust in reliable robots and when losing trust in robots lead to failures.
(A7) *Learning Systems versus Future Everyday Domestic Life: A Designer’s Interpretation of Social Practice Imaginaries* . While smart home technologies promise to adjust to the unique preferences and circumstances of their users, these promises are often at odds with the complexity and unpredictability of everyday domestic life. Viaene et al., draw on the Social Practice Imaginaries method to investigate how automation may support, complicate, or even disrupt the dynamic nature of domestic practices. This exploration enables designers to elicit critical reflection and anticipate issues related to the crisis of routine in the domestic context.

As highlighted in this editorial, this special issue has brought together researchers from various disciplines and application areas to synthesize their perspectives and work that investigate the aspect of respecting human autonomy through approaches of human-centered AI. We expect that this collection informs future research and AI innovation with considerations on how to respect human autonomy.

